# Annotations

**Published:** 1905-02-18

**Authors:** 


### Annotations.

### Public Prayer and Typhoid Fever.

The Bishop of Lincoln has compiled a special prayer for use in the churches at Lincoln, with reference to the epidemic of typhoid fever which is prevalent in the cathedral city. From the Bishop's point of view his action is entirely commendable, but we hope that the Corporation will not consider that it in any way relieves them from the necessity of prosecuting the most vigorous measures for the purpose of arresting the spread of the outbreak. The Bishop, of course, exhorts his clergy and the citizens to ask the Almighty that the sickness may be assuaged; it is for the local authorities to see that practical steps are taken in order that this result may be achieved. There is no doubt that the present position is serious. According to the official statement, there were nearly 600 cases from December up to Tuesday night, and the distress is sufficiently great to have caused the Mayor to start a relief fund. To meet the emergency, several auxiliary hospitals have been opened, including beds in the Drill Hall for nearly 100 patients. With regard to the origin of the epidemic, the discovery by Dr. Klein, the Local Government Board analyst, of typhoid bacilli in a sample of water from the municipal supply, seems to be conclusive, and the Mayor admits that the supply is "only of second-class quality." About 10 years ago the town of King's Lynn suffered from a similar visitation, and then, as now, the inhabitants refused to drink the water—when the disease had made good its footing. So far the mortality at Lincoln has not been so high as it was at Lynn, but it is impossible to say what the developments may be. The number of deaths is stated to be under 30, and the victims include the sanitary inspector of the city. It is all very well, and very desirable, to offer up prayer for the recovery of the suffering and the abatement of the scourge; but if the Corporation of Lincoln had only taken the simple precaution of regularly testing the purity of their water, probably there would have been no epidemic.

### A Municipal Milk Supply.

The advice of Dr. G. F. McCleary in his address to the Association of Medical Officers of Health on "Reform of the Milk Supply" will doubtless commend itself to those who believe in the policy of "thorough" as well as to adherents of the doctrine that those who wish a thing well done must do it themselves. In a word Dr. McCleary's conclusion is that if towns want pure milk they must get if for themselves instead of depending on outside bodies to get it for them. To this the critics of municipal trading will doubtless have something to say, yet it cannot be denied that pure milk is as necessary to the public health as pure water and even more necessary than electric tramways. Under existing arrangements official control comes into operation too late in the day. Milk or other food stuff may be inspected on its arrival in our large towns but the sources of supply are for the most part outside the municipal authority. And it is here that the most serious risks are encountered. What is to be desired is, first, prevention of pollution at the source of supply; secondly, protection from the risk of contamination during transit and distribution; and thirdly, sale of the milk at a cost which makes it obtainable by the poorer members of the community. What is wanted is such a continuous control as will secure distribution of the milk in the pure state it is yielded by healthy and well-kept cows. Possibly this is attained in some cases by private enterprise, but even when well organised this, as a recent case in the law Courts has shown, may fail of success. Official authority if properly supported would, it is urged, prove more effective. That its adoption should be urged by those whose work introduces them to the private and public mischiefs which follow the introduction of impure milk into a community is not without significance. The question is a practical one, and is not to be decided by appeals to economic theory.

### Pharmacists and the Pharmacopœia.

In the daily life and work of the pharmacist the National Pharmacopœia exercises an influence at once constant and commanding, and it is therefore hardly a matter for wonder that pharmacists should desire to play some part in the construction of that volume. From its demands and control they cannot escape, and it is natural they should wish to be heard regarding the regulations which govern so much of their work. The question whether this ambition should or should not be gratified may, in our opinion, be determined by a very simple test—namely, the welfare of the public. It is too often forgotten that the Pharmacopœia exists not for the convenience of prescribers or dispensers, but in the general interest of the community. By providing an authoritative standard it secures qualitative and quantitative uniformity in the interpretation of physicians' prescriptions, and so protects the public from the dangers attached to the use of different standards according to the whim of individual pharmacists. Whatever enables the Pharmacopœia successfully to achieve this function may reasonably claim to be in the public interest, and it is on this ground, and not because of any inherent right, that we sympathise with the aspirations of the pharmacists to take some part in the compilation of the official list of remedies. The extent of such co-operation has recently been well defined by the chairman of the Pharmacopœia Committee. Whilst Dr. MacAlister rightly insists that it lies with the doctors to say what remedies should be included in the pharmacopœia and what must be the strengths of their several preparations it is the pharmacists who are best able to define the technical processes of chemical and galenical pharmacy. It is by a combination of the two forces that the desired end will be attained. The pharmacists, in addition, are able to provide information regarding the frequency or otherwise with which the various remedies are prescribed. This is a matter of considerable importance seeing that the Pharmacopœia exists not so much to indicate what medicines are useful as to define those that are used. Whatever substances are more or less frequently ordered by physicians need official description in the public interest and ought therefore to be included in the official list. On this point as well as in respect to the technicalities of their art the pharmacists can render effective service to the Pharmacopœia Committee.

